# Ethnotaxonomy of birds by the inhabitants of Pedra Branca Village, Santa Teresinha municipality, Bahia state, Brazil

**DOI:** 10.1186/1746-4269-10-55

**Published:** 2014-07-10

**Authors:** Ana Teresa Galvagne Loss, Eraldo Medeiros Costa Neto, Caio Graco Machado, Fernando Moreira Flores

**Affiliations:** 1 Post-Graduation Program in Zoology, Department of Biological Sciences, Feira de Santana State University, Feira de Santana, Bahia 44036-900, Brazil; 2 Department of Biological Sciences, Feira de Santana State University, Feira de Santana, Bahia, Brazil

**Keywords:** Human beings, Bird, Ethno-ornithology, Local names, Hierarchical levels, Vocalization, Ethnoetymology

## Abstract

**Background:**

Studies on popular names of birds help to understand the relationship between human beings and birds and it also contributes to the field of ornithology.

**Methods:**

This study aims to register the ethnotaxonomy of birds in the village of Pedra Branca, Santa Teresinha municipality, Bahia State, Brazil, by cataloguing and identifying their popular names, besides understanding the ethnoclassification system of local bird species. The ethno-ornithological data were obtained by means of semi-structured open interviews, and projective tests.

**Results:**

We interviewed 48 residents and, in order to identify species, we chose five informants with a more detailed knowledge on local avifauna. We registered 139 common names, distributed into 108 ethnospecies and 33 synonyms, referring to 117 species. Nomenclatural criteria more frequently used were vocalization and coloring patterns. Following Berlin’s principles of ethnobiological classification, three hierarchical levels were registered: life form, generic and specific, with three types of correspondence between Linnaean and folk classification systems. The bird life form (“pássaro” in Portuguese) was associated only to wild species.

**Conclusions:**

The ethno-ornithological research in Pedra Branca Village has contributed with new information on popular nomenclature of birds and their etymology, showing that folk knowledge on birds is conveyed within the community.

## Introduction

Studies on biological ethnotaxonomy aim at investigating how living organisms are perceived, identified, named and classified, seeking to understand how people categorize (ethnosemantic domains) and organize (ethnotaxonomic structures) nature elements [1-4]. Some ethnobiological principles of classification and nomenclature aim to identify similarities between cognitive systems in various societies. So, it is important to find out what are the ethnoclassification criteria (morphological, ecological, ethological, etc.), in order to develop a representative taxonomy of the classification system within a certain community [5,4-8]. Ethnobiological classification may be a good indicator of the cognitive and behavioral language process [9].

Considering that human beings, on different parts of the world, use similar cognitive strategies to classify living things and organize biological concepts, studies on ethnotaxonomy show, in fact, that the main problem has always been finding what are the similarities or differences that could be really important for classification purposes [10-13].

Among current animal species, birds draw attention due to their gorgeous coloring and shrill songs [14]. Cooker [15] registered the names attributed by the Chippewa Indians, in northern Mexico, to birds from their region, reporting those they used; this investigation resulted in the first ethno-ornithological study, titled *Bird nomenclature of the Chippewa Indians*. Regarding the origin of names, the author observed the use of morphological criteria and habitats, and there were also names of species with no meaning.

Whereas the scientific nomenclature of birds has been established for over 200years, popular and vernacular designation, as a product of people’s imagination, has no systematization. Vernacular names are popular, vulgar, or common names, which are names adopted by people who live in the regions inhabited by birds [14]. Thus, investigating vernacular names of birds from a certain region provides ornithology with the possibility of registering new occurrences, describing unknown behaviors, locating endangered species, and pointing out conservation alternatives, as well as understanding the relationships between human beings and birds, explaining to society the intrinsic value of cultural diversity [16].

In Brazil, investigations and contributions from local knowledge on birds started when settlers first came to Brazil; they registered bird popular names, as well as stories and legends told by native people, which served, since the beginning, as data on the Brazilian avifauna [7]. Later, many studies were conducted registering popular names and the reproductive ecology of several species [17]; descriptions of customs, superstitions, and Brazilian and American legends, addressing the etymology of some names [18]. Sick [14], who is a reference in ornithology, deal with vocalization transcripts, etymology of bird names, and in a very summarized way, legends involving some species.

Guided by a theoretical framework of cognitive anthropology, there are the following works: Jensen [19] examined bird classification systems among four Indian groups having similar environments and lifestyles in the Amazon: Wayampi, Urubu-Ka’apor, Sateré-Mawé, and Apalaí; Giannini [20] investigated, along with Xicrin Indians, the existence of an Indian ethnoclassification of the avifauna from the Rio Cateté region, in Pará, Brazil; and Carrara [21] examined the ethnobiological classification of Xavante Indians in Mato Grosso, Brazil.

The present article registers the ethnotaxonomy of birds known by residents of the village of Pedra Branca, in the municipality of Santa Teresinha, Bahia, Brazil, cataloguing and identifying popular names, with their etymological description, besides analyzing the ethnoclassification system of local bird species. The biological ethnotaxonomic study is of great importance both for understanding and grasping local biodiversity and investigating the universality of human ability to classify the biological world.

## Materials and methods

### Study area

**Figure 1:**
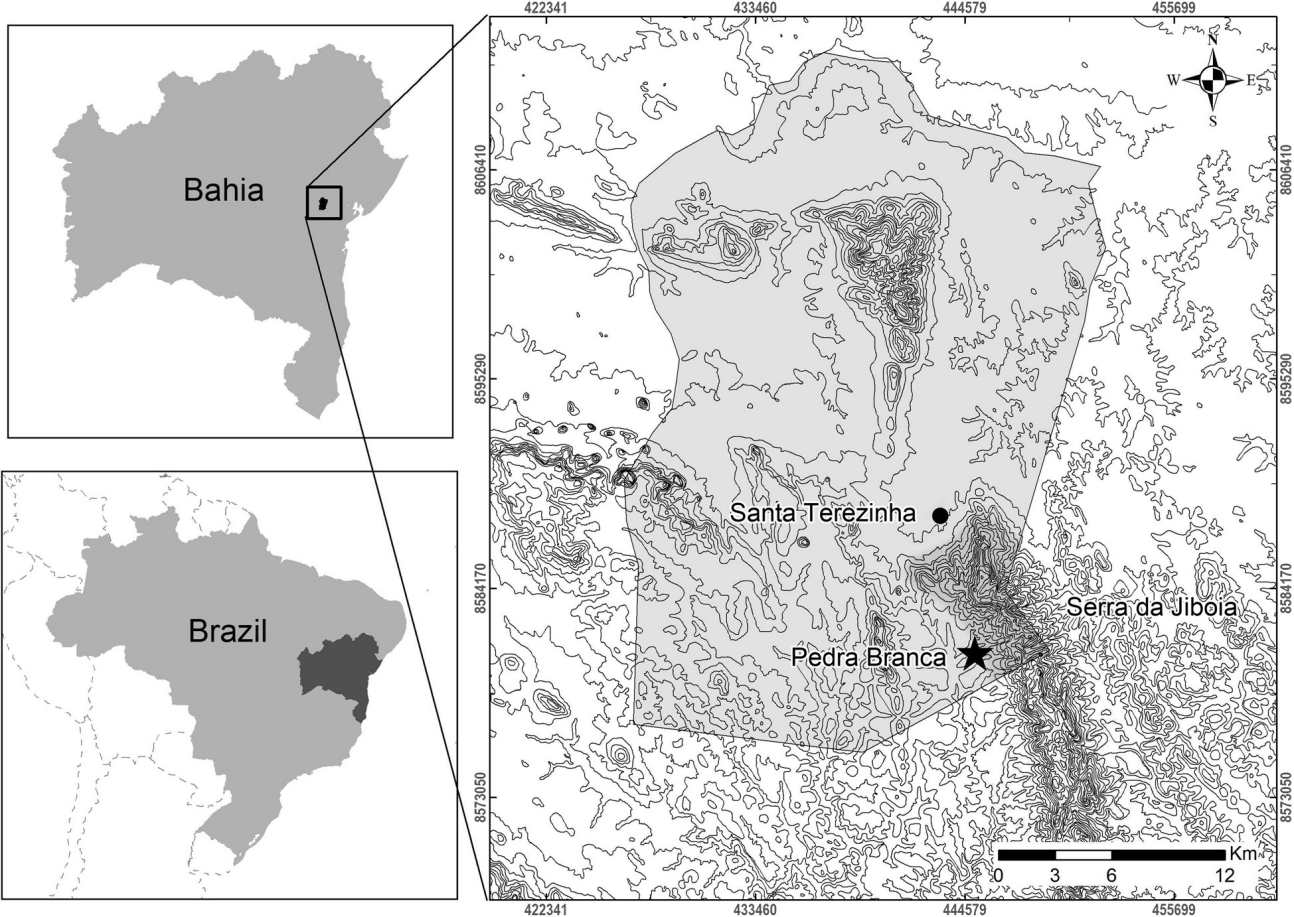
Location of the village of Pedra Branca (Santa Teresinha, Bahia) and Serra da Jiboia.

The village of Pedra Branca is located in the municipality of Santa Teresinha (12°44’30”S and 39°34’50”W), in the central-west area of Bahia State, Brazil, a region with sub-humid to dry climate features (Figure 1). This municipality has a population of 9,648 people and it is 192 km far from the state capital city, Salvador [22,23].

Pedra Branca has 406 residents, with 136 families enrolled in the local health care unit and higher concentrations in the age groups from 20 to 39 years and above 60 years. Local agriculture is based on cassava crop (*Manihot esculenta* Crantz, Euphorbiaceae) and grape crop for producing red wine; livestock is related to cattle breeding. Men sometimes have jobs in civil construction [24,25,23].

The village lies at the bottom of a mountainous massive known as Serra da Jiboia, which has around 22,500 ha in area and a maximum altitude of 850 m. Serra da Jiboia is located southern Recôncavo region in Bahia and comprehends the territories of five municipalities: Castro Alves, Elísio Medrado, Santa Teresinha, São Miguel das Matas, and Varzedo [26-28].

Serra da Jiboia is located in an ecotone zone, between the ecosystems of Atlantic Forest and Caatinga, which provides it with a great diversity of climates, reliefs, soils, vegetation, and fauna, being one of the most western sites of the Atlantic Forest in Bahia and one of the wettest forests in the most northern hillside of the state. Its climate ranges from humid tropical, at east and southeast, to sub-humid tropical, at west and northwest [28]. Floristic studies in the area report the occurrence of many plant formations with rock fields on the top, Caatinga at the bottom, and hygrophilous forest at the slopes [29-32]. Regarding avifauna, two studies are known for the region, on distinct areas of Serra da Jiboia, where 221 species were registered by Freitas and Moraes [33] and 233 species were registered in surveys conducted by the Feira de Santana State University (UEFS) (PhD Caio Graco Machado, person. commun.). The richest region comprises areas where Pedra Branca inhabitants perform some of their daily activities.

### Data collection and analysis

Field collection was conducted within the period from August 2011 to June 2012 and 48 residents of both sexes were interviewed, 24 men and 24 women, aged from 18 to 87 years. A free and informed consent term (Resolution 196/1996 from the Ministry of Health) has been prepared to explain the objectives of this study; it was distributed to participants, asking if they agreed to provide information, respecting the decision of those who declined to participate in the research. The study was approved by the Research Ethics Committee of UEFS (CAAE 0077.059.000-11).

Initially, data were obtained both through open interviews, exploring and detailing the theme under study, and semi-structured interviews based on a list of pre-selected topics. As a final phase of data collection, a projective test was performed consisting on the presentation of visual and auditory resources to encourage interviewees to speak spontaneously about what they saw and heard [34]. These photographs and vocalizations were of species of birds that inhabit both Atlantic Forest and Caatinga environs. The photographs used came from personal files and Wikiaves [35], while the auditory resources came from Wikiaves’ data bank. Five male informants (ages 31–63), who showed a more detailed knowledge on the local avifauna during the interviews, were chosen for this data collecting technique. They were approached individually and were very skillful on identifying bird species.

The term ethnospecies is employed in this study when an ethnobiological taxonomic category corresponds to the Linnaean scientific species, independently of how many popular names it receives. The scientific nomenclature followed the Comitê Brasileiro de Registros Ornitológicos [36]. The endangered status followed the Lista Vermelha das Espécies Ameaçadas do Ministério do Meio Ambiente [37], the International Union of Conservation of Nature [38], and the Birdlife International [39].

Data were analyzed using the union model. According to this model, all available information on the surveyed subject is to be considered [40]. For checking their reliability, interviews were conducted both in synchronous situations, with the same question being asked to different individuals in a short interval of time, and diachronic situations, when the same question was asked to the same individual in a long time interval [41].

All ethnographic materials (recordings, transcripts, photographs, and drawings) are kept at the Laboratory of Ethnobiology and Ethnoecology of the Universidade Estadual de Feira de Santana (UEFS), state of Bahia, for evidential purposes.

This study adopted the hierarchy structure proposed by Berlin [4], whose decreasing inclusion taxonomic classes form the following levels: kingdom, life form, intermediate, generic, specific, and variety. We used Venn’s diagram [42,43] for relating the ethnobiological taxonomy to the Linnaean taxonomy,, where there is indication of Linnaean and ethnobiological taxa by means of circles with different marks, and it also enables showing the closeness between folk members [4]. For comparing the popular taxonomy to the Linnaean taxonomy, we used correspondence categories in evaluations regarding the popular generic names and scientific species, such as 1:1 correspondence, where the popular generic name refers to a single scientific species; over-differentiation, when two or more generic taxa refer to a scientific species; and under-differentiation may have two types, type 1 occurs when a single generic name refers to two or more species from the same scientific genus and type 2 occurs when a single generic name refers to two or more species from different scientific genera [44].

## Results and discussion

### Name formation and identification of ethnospecies

The interviewed cited 139 common names of wild birds, which refer to 117 Linnaean species (Table 1). Unlike the Berlinean system [4], when the author says that the name structure of specific taxa in the ethnobiological classification systems is regularly binomial, out of the total number of common names in this study, 63 have binomial names and 77 have monomial names. See Table 2 to check out the glossary of all local bird names.

**Table 1:** List of birds’ common names and their respective academic correspondences

**Local common names**	**Synonymy**	**Scientific names**	**Family**
Acauã	Cauã	*Herpetotheres cachinnans* (Linnaeus, 1758)	Falconidae Leach, 1820
Alma-de-gato		*Piaya cayana* (Linnaeus, 1766)	Cuculidae Swainson, 1837
Andorinha		*Progne tapera* (Vieillot, 1817)	Hirundinidae Rafinesque, 1815
Anu-branco		*Guira guira* (Gmelin, 1788)	Cuculidae Swainson, 1837
Anu-preto		*Crotophaga ani* Linnaeus, 1758	Cuculidae Swainson, 1837
Aracuã		*Ortalis guttata* (Spix, 1825)	Cracidae Rafinesque, 1815
Araponga		*Procnias nudicollis* (Vieillot, 1817)	Cotingidae Bonaparte, 1849
Assanhaço-comum		*Tangara sayaca* (Linnaeus, 1766)	Thraupidae Cabanis, 1847
Assanhaço-coqueiro		*Tangara palmarum* (Wied, 1823)	Thraupidae Cabanis, 1847
Azulão		*Cyanoloxia brissonii* (Lichtenstein, 1823)	Cardinalidae Ridgway, 1901
Azuzinho		*Tersina viridis* (Illiger, 1811)	Thraupidae Cabanis, 1847
Beija-flor		*Phaethornis pretrei* (Lesson & Delattre, 1839)	Trochilidae Vigors, 1825
		*Florisuga fusca* (Vieillot, 1817)	Trochilidae Vigors, 1825
Beija-flor-rabo-de-tesoura		*Eupetomena macroura* (Gmelin, 1788)	Trochilidae Vigors, 1825
Beija-flor-verde	Martim-pescador	*Galbula ruficauda* Cuvier, 1816	Galbulidae Vigors, 1825
Bem-te-vi	Bem-te-vi-coroão	*Pitangus sulphuratus* (Linnaeus, 1766)	Tyrannidae Vigors, 1825
Bem-te-vi-ciseri		*Megarynchus pitanguá* (Linnaeus, 1766)	Tyrannidae Vigors, 1825
Bem-te-vi-menor		*Myiozetetes similis *(Spix, 1825)	Tyrannidae Vigors, 1825
Bico-de-lacre		*Estrilda astrild* (Linnaeus, 1758)	Estrildidae Bonaparte, 1850
Bigode		*Sporophila lineola* (Linnaeus, 1758)	Emberezidae Vigors, 1825
Caboculinho		*Sporophila bouvreuil* (Statius Muller, 1776)	Emberezidae Vigors, 1825
Caburé		*Glaucidium brasilianu* (Gmelin, 1788)	Strigidae Leach, 1820
Caburé-de-estaca	Caburé-de-murundu	*Athene cunicularia* (Molina, 1782)	Strigidae Leach, 1820
Caga-cebo		*Todirostrum cinereum* (Linnaeus, 1766)	Tyrannoidea Vigors, 1825
Canário-belga	Canário-da-Alemanha	*Serinus canária* (Linnaeus, 1758)	Fringillidae Leach, 1820
Canário-da-capoura		*Sicalis luteola* (Sparrman, 1789)	Emberezidae Vigors, 1825
Canário-da-terra	Canário-comum	*Sicalis flaveola* (Linnaeus, 1766)	Emberezidae Vigors, 1825
Cancan		*Cyanocorax cyanopogon* (Wied, 1821)	Corvidae Leach, 1820
Capitão-de-preá		*Laterallus viridis* (Statius Muller, 1776)	Rallidae Rafinesque, 1815
Cardeal		*Paroaria dominicana* (Linnaeus, 1758)	Thraupidae Cabanis, 1847
Carrega-madeira	Gué-gué	*Phacellodomus rufifrons* (Wied, 1821)	Furnariidae Gray, 1840
Casaca-de-couro	Pica-pau	*Pseudoseisura cristata* (Spix, 1824)	Furnariidae Gray, 1840
Cava-chão		*Nystalus maculatus* (Gmelin, 1788)	Bucconidae Horsfield, 1821
Chapéu-de-couro		*Chrysomus ruficapillus* (Vieillot, 1819)	Icteridae Vigors, 1825
Charuteira		*Gallinago paraguaiae* (Vieillot, 1816)	Scolopacidae Rafinesque, 1815
Chorão		*Sporophila leucoptera* (Vieillot, 1817)	Emberezidae Vigors, 1825
Chupa-laranja	Papa-laranja	*Coereba flaveola* (Linnaeus, 1758)	Coerebidae D'orbigny & Lafresnaye, 1838
Codorna-pimpão	Codorna-maior	*Nothura maculosa* (Temminck, 1815)	Tinamidae Gray, 1840
Codorna-piriri		*Nothura boraquira* (Spix, 1825)	Tinamidae Gray, 1840
Coleiro		*Sporophila albogularis* (Spix, 1825)	Emberezidae Vigors, 1825
Corró		*Taraba major* (Vieillot, 1816)	Thamnophilidae Swainson, 1824
Corró-pequeno		*Thamnophilus pelzelni* Hellmayr, 1924	Thamnophilidae Swainson, 1824
Corta-colete		*Tangara cayana* (Linnaeus, 1766)	Thraupidae Cabanis, 1847
Coruja	Coruja-amanhã-eu-vou; Coruja-bacurau; Bacurau	*Hydropsalis albicollis (*Gmelin, 1789)	Caprimulgidae Vigors, 1825
Corujão		*Tyto alba* (Scopoli, 1769)	Tytonidae Mathews, 1912
		*Pulsatrix koeniswaldiana* (Bertoni & Bertoni, 1901)	Strigidae Leach, 1820
		*Stix virgata* (Cassin, 1849)	Strigidae Leach, 1820
Corujão-de-orelha	Caburé-de-orelha	*Megascops choliba*	Strigidae
		(Vieillot, 1817)	Leach, 1820
Coruja-rabo-de-tesoura	Coruja-tô-rica	*Hydropsalis torquata (*Gmelin, 1789)	Caprimulgidae Vigors, 1825
Cuiuba		*Forpus xanthopterygius *(Spix, 1824)	Psittacidae Rafinesque, 1815
Curió		*Sporophila angolensis* (Linnaeus, 1766)	Emberezidae Vigors, 1825
Espanta-boiada	Quero-quero	*Vanellus chilensis* (Molina, 1782)	Charadriidae Leach, 1820
Estevo	Trinca-ferro, Pixarro; Vaqueiro	*Saltator similis* d'Orbigny & Lafresnaye, 1837	Thraupidae Cabanis, 1847
Estrelinha		*Lanio pileatus *(Wied, 1821)	Thraupidae Cabanis, 1847
Garça		*Ardea Alba* Linnaeus, 1758	Ardeidae Leach, 1820
		*Bubulcus íbis *(Linnaeus, 1758)	Ardeidae Leach, 1820
Garrincha		*Troglodytes musculus* Naumann, 1823	Troglodytidae Swainson, 1831
Gavião-carcará		*Caracara plancus (*Miller, 1777)	Falconidae Leach, 1820
Gavião-carrapateiro	Carcará-pequeno	*Milvago chimachima* (Vieillot, 1816)	Falconidae Leach, 1820
Gavião-pé-de-morro		*Geranospiza caerulescens* (Vieillot, 1817)	Accipitridae Vigors, 1824
Gavião-pedrez	Gavião-pega-pinto	*Rupornis magnirostris* (Gmelin, 1788)	Accipitridae Vigors, 1824
Gavião-peneira		*Elanus leucurus* (Vieillot, 1818)	Accipitridae Vigors, 1824
Gavião-rapina		*Geranoaetus albicaudatus* (Vieillot, 1816)	Accipitridae Vigors, 1824
		*Buteo brachyurus* Vieillot, 1816	Accipitridae Vigors, 1824
Guriatá-verdadeira		*Euphonia violácea* (Linnaeus, 1758)	Fringillidae Leach, 1820
Guriatá-vivi		*Euphonia clorotica* (Linnaeus, 1766)	Fringillidae Leach, 1820
Jacu-verdadeiro	Jacu-gogó-vermelho; Jacu-pemba	*Penelope superciliaris* Temminck, 1815	Cracidae Rafinesque, 1815
Jesus-meu-Deus		*Zonotrichia capensis* (Statius Muller, 1776)	Emberezidae Vigors, 1825
João-de-barro		*Furnarius rufus* (Gmelin, 1788)	Furnariidae Gray, 1840
Juriti		*Leptotila verreauxi* Bonaparte, 1855	Columbidae Leach, 1820
Lavandeira		*Fluvicola negenta* (Linnaeus, 1766)	Tyrannidae Vigors, 1825
Macuca		*Tinamus solitarius* (Vieillot, 1819)	Tinamidae Gray, 1840
Mãe-da-lua	Urutau	*Nyctibius griséus* (Gmelin, 1789)	Nyctibiidae Chenu & Des Murs, 1851
Maria-do-dia		*Elaenia flavogaster* (Thunberg, 1822)	Tyrannidae Vigors, 1825
Marreca	Pato-verdadeiro; Pato-d’água	*Porphyrio Martinica* (Linnaeus, 1766)	Rallidae Rafinesque, 1815
		*Dendrocygna viduata* (Linnaeus, 1766)	Anatidae Leach, 1820
Martim-pescador		*Megaceryle torquata* (Linnaeus, 1766)	Alcedinidae Rafinesque, 1815
Mergulhão		*Tachybaptus dominicus* (Linnaeus, 1766)	Podicipedidae Bonaparte, 1831
Nambu-pé-roxo		*Crypturellus tataupa* (Temminck, 1815)	Tinamidae Gray, 1840
Nambu-pé-vermelho		*Crypturellus parvirostris* (Wagler, 1827)	Tinamidae Gray, 1840
Papa-arroz	Sangue-de-boi	*Sturnella superciliaris* (Bonaparte, 1850)	Icteridae Vigors, 1825
Papa-café		*Schistochlamys ruficapillus* (Vieillot, 1817)	Thraupidae Cabanis, 1847
Papa-capim		*Sporophila nigricollis* (Vieillot, 1823)	Emberezidae Vigors, 1825
Pardal		*Passer domesticus* (Linnaeus, 1758)	Passeridae Rafinesque, 1815
Pássaro-preto		*Gnorimopsar chopi* (Vieillot, 1819)	Icteridae Vigors, 1825
Pêga		*Icteru pyrrhopterus* (Vieillot, 1819)	Icteridae Vigors, 1825
Peixe-frito	Sede-sede	*Tapera naevia* (Linnaeus, 1766)	Taperinae Verheyen, 1956
Perdiz		*Rhynchotus rufescens* (Temminck, 1815)	Tinamidae Gray, 1840
Periquito		*Aratinga cactorum* (Kuhl, 1820)	Psittacidae Rafinesque, 1815
Pica-pau		*Veniliornis passerines* (Linnaeus, 1766)	Picidae Leach, 1820
		*Drycopus lineatus* (Linnaeus, 1766)	Picidae Leach, 1820
		*Colaptes melanochloros *(Gmelin, 1788)	Picidae Leach, 1820
Pintassilgo		*Sporagra yarrellii* (Audubon, 1839)	Fringillidae Leach, 1820
Pomba-verdadeira	Pomba-do-sertão	*Patagioenas picazuro* (Temminck, 1813)	Columbidae Leach, 1820
Pomba-do-Pará		Não identificado	-
Rolinha-branca		*Columbina picui* (Temminck, 1813)	Columbidae Leach, 1820
Rolinha-caldo-de-feijão		*Columbina talpacoti (*Temminck, 1811)	Columbidae Leach, 1820
Rolinha-fogo-pago		*Columbina squamatta* (Lesson, 1831)	Columbidae Leach, 1820
Rolinha-Santo-Antônio		*Columbina minuta* (Linnaeus, 1766)	Columbidae Leach, 1820
Sabiá-bico-de-osso		*Turdus amaurochalinus* Cabanis, 1850	Turdidae Rafinesque, 1815
Sabiá-branca		*Turdus leucomela* Vieillot, 1818	Turdidae Rafinesque, 1815
Sabiá-coca		*Turdus rufiventris* Vieillot, 1818	Turdidae Rafinesque, 1815
Sabiá-lasca-carne		*Mimus saturninus* (Lichtenstein, 1823)	Mimidae Bonaparte, 1853
Saiacaia	Cavala	*Gallinago undulata* (Boddaert, 1783)	Scolopacidae Rafinesque, 1815
Sangue-de-boi		*Ramphocelus bresilius* (Linnaeus, 1766)	*Thraupidae* Cabanis, 1847
Saracura	Três-potes; Sete-potes	*Aramides cajanea* (Statius Muller, 1776)	Rallidae Rafinesque, 1815
Siriema		*Cariama cristata* (Linnaeus, 1766)	Cariamidae Bonaparte, 1850
Socó-boi		*Butorides striata (*Linnaeus, 1758)	Ardeidae Leach, 1820
Sofrê		*Icterus jamacaii* (Gmelin, 1788)	Icteridae Vigors, 1825
Tiotoin		*Synallaxis frontalis* Pelzeln, 1859	Furnariidae Gray, 1840
Tiziu	Biziu	*Volatinia jacarina* (Linnaeus, 1766)	Emberezidae Vigors, 1825
Tororó		Não identificado	-
Tucano		*Ramphastus vitellinus* Lichtenstein, 1823	Ramphastidae Vigors, 1825
Urubu-da-cabeça-vermelha	Bosteiro	*Cathartis aurea* (Linnaeus, 1758)	Cathartidae Lafresnaye, 1839
Urubu-preto	Urubu-carniceiro	*Coragips atratus* (Bechstein, 1793)	Cathartidae Lafresnaye, 1839
Urubu-rei		*Sarcoramphus papa* (Linnaeus, 1758)	Cathartidae Lafresnaye, 1839
Viuvinha		*Xolmis irupero* (Vieillot, 1823)	Tyrannidae Vigors, 1825
Xanana		*Jacana jacana* (Linnaeus, 1766)	Jacanidae Chenu & Des Murs, 1854
Zabelê		*Crypturellus noctivagus* (Wied, 1820)	Tinamidae Gray, 1840

Common names follow the local language.

*Endangered species [37].

**Table 2:** Correspondence between bird species common names and scientific names

**Local common names**	**English common names**	**Espécies científicas**
Acauã	Laughing Falcon*	*Herpetotheres cachinnans* (Linnaeus, 1758)
Alma-de-gato	Squirrel Cuckoo*	*Piaya cayana* (Linnaeus, 1766)
Andorinha	Brown-chested Martin*	*Progne tapera* (Vieillot, 1817)
Anu-branco	White Anu**	*Guira guira* (Gmelin, 1788)
Anu-preto	Black Anu**	*Crotophaga ani* Linnaeus, 1758
Aracuã	Speckled Chachalaca*	*Ortalis guttata* (Spix, 1825)
Araponga	Bare-throated Bellbird*	*Procnias nudicollis* (Vieillot, 1817)
Assanhaço-comum	Sayaca Tanager*	*Tangara sayaca* (Linnaeus, 1766)
Assanhaço-coqueiro	Coconut Tanager**	*Tangara palmarum* (Wied, 1823)
Azulão	Big Blue**	*Cyanoloxia brissonii* (Lichtenstein, 1823)
Azuzinho	Little Blue**	*Tersina viridis* (Illiger, 1811)
Bacurau	Night Hawk**	*Hydropsalis albicollis* (Gmelin, 1789)
Beija-flor	Planalto Hermit*	*Phaethornis pretrei* (Lesson & Delattre, 1839)
	Black Jacobin*	*Florisuga fusca* (Vieillot, 1817)
Beija-flor-rabo-de-tesoura	Scissor-tailed Hummingbird**	*Eupetomena macroura* (Gmelin, 1788)
Beija-flor-verde	Green Hummingbird**	*Galbula ruficauda* Cuvier, 1816
Bem-te-vi	Great Kiskadee*	*Pitangus sulphuratus* (Linnaeus, 1766)
Bem-te-vi-ciseri	Kiskadee**	*Megarynchus pitanguá* (Linnaeus, 1766)
Bem-te-vi-coroão	Crowned Kiskadee**	*Pitangus sulphuratus* (Linnaeus, 1766)
Bem-te-vi-menor	Small Kiskadee**	*Myiozetetes similis* (Spix, 1825)
Bico-de-lacre	Common Waxbill*	*Estrilda astrild* (Linnaeus, 1758)
Bigode	Mustache**	*Sporophila lineola* (Linnaeus, 1758)
Biziu	Blue-black Grassquit*	*Volatinia jacarina* (Linnaeus, 1766)
Bosteiro	Shit-eater**	*Cathartis aurea* (Linnaeus, 1758)
Caboculinho	Copper Seedeater*	*Sporophila bouvreuil* (Statius Muller, 1776)
Caburé	Ferruginous Pygmy-Owl*	*Glaucidium brasilianu* (Gmelin, 1788)
Caburé-de-estaca	Pole Pygmy-Owl**	*Athene cunicularia* (Molina, 1782)
Caburé-de-murundu	Hillock Pygmy-Owl**	*Athene cunicularia* (Molina, 1782)
Caburé-de-orelha	Eared Pygmy-Owl**	*Megascops choliba* (Vieillot, 1817)
Caga-cebo	Common Tody-Flycatcher*	*Todirostrum cinereum* (Linnaeus, 1766)
Canário-belga	Belgian Canary**	*Serinus canária* (Linnaeus, 1758)
Canário-comum	Saffron Finch*	*Sicalis flaveola* (Linnaeus, 1766)
Canário-da-Alemanha	German Canary**	*Serinus canária* (Linnaeus, 1758)
Canário-da-capoura	Barton Canary**	*Sicalis luteola* (Sparrman, 1789)
Canário-da-terra	Earthy Canary**	*Sicalis flaveola* (Linnaeus, 1766)
Cancan	White-naped Jay*	*Cyanocorax cyanopogon* (Wied, 1821)
Capitão-de-preá	Russet-crowned Crake*	*Laterallus viridis* (Statius Muller, 1776)
Cardeal	Red-cowled Cardinal*	*Paroaria dominicana* (Linnaeus, 1758)
Carrega-madeira	Loading timber**	*Phacellodomus rufifrons* (Wied, 1821)
Casaca-de-couro	Leather jacket**	*Pseudoseisura cristata* (Spix, 1824)
Cava-chão	Floor Digger**	*Nystalus maculatus* (Gmelin, 1788)
Cavala	Mackerel**	*Gallinago undulata* (Boddaert, 1783)
Chapéu-de-couro	Chestnut-capped Blackbird*	*Chrysomus ruficapillus* (Vieillot, 1819)
Charuteira	Cigarette Case**	*Gallinago paraguaiae* (Vieillot, 1816)
Chorão	Crybaby**	*Sporophila leucoptera* (Vieillot, 1817)
Chupa-laranja	Orange Sucker**	*Coereba flaveola* (Linnaeus, 1758)
Codorna-maior	Large Quail**	*Nothura maculosa* (Temminck, 1815)
Codorna-pimpão	Crucian Quail**	*Nothura maculosa* (Temminck, 1815)
Codorna-piriri	Piriri Quail**	*Nothura boraquira* (Spix, 1825)
Coleiro	Collared Seedeater**	*Sporophila albogularis* (Spix, 1825)
Corró	Great Antshrike*	*Taraba major* (Vieillot, 1816)
Corró-pequeno	Little Antshrike**	*Thamnophilus pelzelni* Hellmayr, 1924
Corta-colete	Burnished-buff Tanager*	*Tangara cayana* (Linnaeus, 1766)
Coruja	Owl**	*Hydropsalis albicollis (*Gmelin, 1789)
Coruja-amanhã-eu-vou	Tomorrow-I-will Owl**	*Hydropsalis albicollis (*Gmelin, 1789)
Coruja-bacurau	Night Hawk Owl**	*Hydropsalis albicollis (*Gmelin, 1789)
Corujão	Big Owl**	*Tyto alba* (Scopoli, 1769)
		*Pulsatrix koeniswaldiana* (Bertoni & Bertoni, 1901)
		*Stix virgata* (Cassin, 1849)
Corujão-de-orelha	Eared Owl**	*Megascops choliba* (Vieillot, 1817)
Coruja-rabo-de-tesoura	Scissor-tailed Owl**	*Hydropsalis torquata (*Gmelin, 1789)
Coruja-tô-rica	Wealthy Owl**	*Hydropsalis torquata (*Gmelin, 1789)
Cuiuba	Blue-winged Parrotlet*	*Forpus xanthopterygius* (Spix, 1824)
Curió	Chestnut-bellied Seed-Finch*	*Sporophila angolensis* (Linnaeus, 1766)
Espanta-boiada	Cattle Bugaboo**	*Vanellus chilensis* (Molina, 1782)
Estevo	Green-winged Saltator*	*Saltator similis* d'Orbigny & Lafresnaye, 1837
Estrelinha	Pileated Finch*	*Lanio pileatus* (Wied, 1821)
Garça	Great Egret*	*Ardea Alba* Linnaeus, 1758
	Cattle Egret*	*Bubulcus íbis*(Linnaeus, 1758)
Garrincha	Southern House Wren*	*Troglodytes musculus* Naumann, 1823
Gavião-carcará	Caracara Hawk**	*Caracara plancus* (Miller, 1777)
Gavião-carrapateiro	Tick-eater Hawk**	*Milvago chimachima* (Vieillot, 1816)
Gavião-pé-de-morro	Foot-of-the-hill Hawk**	*Geranospiza caerulescens* (Vieillot, 1817)
Gavião-pedrez	Check Hawk**	*Rupornis magnirostris* (Gmelin, 1788)
Gavião-pega-pinto	Chick-Catcher Hawk**	*Rupornis magnirostris* (Gmelin, 1788)
Gavião-peneira	Strainer Hawk**	*Elanus leucurus* (Vieillot, 1818)
Gavião-rapina	Rapine Hawk**	*Geranoaetus albicaudatus* (Vieillot, 1816) *Buteo brachyurus* Vieillot, 1816
Gué-gué	Rufous-fronted Thornbird*	*Phacellodomus rufifrons* (Wied, 1821)
Guriatá-verdadeira	True Guriatá**	*Euphonia violacea* (Linnaeus, 1758)
Guriatá-vivi	Guriatá**	*Euphonia clorotica* (Linnaeus, 1766)
Jacu-gogó-vermelho	Red throate Guan**	*Penelope superciliaris* Temminck, 1815
Jacu-pemba	Pemba Guan**	*Penelope superciliaris* Temminck, 1815
Jacu-verdadeiro	Rusty-margined Guan*	*Penelope superciliaris T*emminck, 1815
Jesus-meu-Deus	Rufous-collared Sparrow*	*Zonotrichia capensis* (Statius Muller, 1776)
João-de-barro	Rufous Hornero*	*Furnarius rufus* (Gmelin, 1788)
Juriti	White-tipped Dove*	*Leptotila verreauxi* Bonaparte, 1855
Lavandeira	Washer**	*Fluvicola negenta* (Linnaeus, 1766)
Macuca	Solitary Tinamou*	*Tinamus solitarius* (Vieillot, 1819)
Mãe-da-lua	Moon Mother**	*Nyctibius griséus* (Gmelin, 1789)
Maria-do-dia	Day Mary**	*Elaenia flavogaster* (Thunberg, 1822)
Marreca	Purple Gallinule*	*Porphyrio Martinica* (Linnaeus, 1766)
	White-faced Whistling-Duck	*Dendrocygna viduata* (Linnaeus, 1766)
Martim-pescador	Kingfisher**	*Megaceryle torquata* (Linnaeus, 1766)
Martim-pescador	Kingfisher**	*Galbula ruficauda* Cuvier, 1816
Mergulhão	Diver**	*Tachybaptus dominicus* (Linnaeus, 1766)
Nambu-pé-roxo	Purple-foot Tinamou**	*Crypturellus tataupa* (Temminck, 1815)
Nambu-pé-vermelho	Red-foot Tinamou**	*Crypturellus parvirostris* (Wagler, 1827)
Papa-arroz	Rice-eater**	*Sturnella superciliaris* (Bonaparte, 1850)
Papa-café	Coffee-eater**	*Schistochlamys ruficapillus* (Vieillot, 1817)
Papa-capim	Grass-eater**	*Sporophila nigricollis* (Vieillot, 1823)
Papa-laranja	Orange-eater**	*Coereba flaveola* (Linnaeus, 1758)
Pardal	Sparrow**	*Passer domesticus* (Linnaeus, 1758)
Pássaro-preto	Black Bird**	*Gnorimopsar chopi* (Vieillot, 1819)
Pato-d’água	Water Duck**	*Porphyrio Martinica* (Linnaeus, 1766)
		*Dendrocygna viduata* (Linnaeus, 1766)
Pato-verdadeiro	True Duck**	*Porphyrio Martinica* (Linnaeus, 1766)
		*Dendrocygna viduata* (Linnaeus, 1766)
Pêga	Magpie**	*Icteru pyrrhopterus* (Vieillot, 1819)
Peixe-frito	Fried Fish**	*Tapera naevia* (Linnaeus, 1766)
Perdiz	Red-winged Tinamou*	*Rhynchotus rufescens* (Temminck, 1815)
Periquito	Parakeet**	*Aratinga cactorum* (Kuhl, 1820)
Pica-pau	Woodpecker**	*Veniliornis passerines* (Linnaeus, 1766)
		*Drycopus lineatus* (Linnaeus, 1766)
		*Colaptes melanochloros* (Gmelin, 1788)
		*Pseudoseisura cristata* (Spix, 1824)
Pintassilgo	Yellow-faced Siskin*	*Sporagra yarrellii* (Audubon, 1839)
Pixarro	Green-winged Saltator*	*Saltator similis* d'Orbigny & Lafresnaye, 1837
Pomba-do-Pará	Pará’s Dove**	Não identificado
Pomba-do-sertão	Sertão Dove**	*Patagioenas picazuro* (Temminck, 1813)
Pomba-verdadeira	True Dove**	*Patagioenas picazuro* (Temminck, 1813)
Quero-quero	Want-want**	*Vanellus chilensis* (Molina, 1782)
Rolinha-branca	White Turtledove**	*Columbina picui* (Temminck, 1813)
Rolinha-caldo-de-feijão	Bean soup Turtledove**	*Columbina talpacoti* (Temminck, 1811)
Rolinha-fogo-pago		*Columbina squamatta* (Lesson, 1831)
Rolinha-Santo-Antônio	Saint Antony Turtledove**	*Columbina minuta* (Linnaeus, 1766)
Sabiá-bico-de-osso	Boned-beak Thrush**	*Turdus amaurochalinus* Cabanis, 1850
Sabiá-branca	White Thrush **	*Turdus leucomela* Vieillot, 1818
Sabiá-coca	Coca Thrush**	*Turdus rufiventris* Vieillot, 1818
Sabiá-lasca-carne	Chipping-meat Thrush**	*Mimus saturninus* (Lichtenstein, 1823)
Saiacaia	Skirtfall**	*Gallinago undulata* (Boddaert, 1783)
Sangue-de-boi	Cattle Blood**	*Ramphocelus bresilius* (Linnaeus, 1766)
		*Schistochlamys ruficapillus* (Vieillot, 1817)
Saracura	Gray-necked Wood-Rail*	*Aramides cajanea* (Statius Muller, 1776)
Sede-sede	Thirst-thirst**	*Tapera naevia* (Linnaeus, 1766)
Sete-potes	Seven Pots**	*Aramides cajanea* (Statius Muller, 1776)
Siriema	Red-legged Seriema*	*Cariama cristata* (Linnaeus, 1766)
Socó-boi	Striated Heron*	*Butorides striata* (Linnaeus, 1758)
Sofrê	Sufferer**	*Icterus jamacaii* (Gmelin, 1788)
Tiotoin	Sooty-fronted Spinetail*	*Synallaxis frontalis* Pelzeln, 1859
Tiziu	Blue-black Grassquit*	*Volatinia jacarina* (Linnaeus, 1766)
Tororó	- - -	Not identified
Três-potes	Three Pots**	*Aramides cajanea* (Statius Muller, 1776)
Trinca-ferro	Green-winged Saltator*	*Saltator similis* d'Orbigny & Lafresnaye, 1837
Tucano	Toucan**	*Ramphastus vitellinus* Lichtenstein, 1823
Urubu-carniceiro	Butcher Vulture**	*Cathartis áurea* (Linnaeus, 1758)
Urubu-da-cabeça-vermelha	Red-headed Vulture**	*Cathartis áurea* (Linnaeus, 1758)
Urubu-preto	Black Vulture**	*Coragips atratus* (Bechstein, 1793)
Urubu-rei	King Vulture**	*Sarcoramphus papa* (Linnaeus, 1758)
Urutau	Common Potoo*	*Nyctibius griséus* (Gmelin, 1789)
Vaqueiro	Cowboy**	*Saltator similis* d'Orbigny & Lafresnaye, 1837
Viuvinha	Little Widow**	*Xolmis irupero* (Vieillot, 1823)
Xanana	Wattled Jacana*	*Jacana jacana* (Linnaeus, 1766)
Zabelê	Yellow-legged Tinamou*	*Crypturellus noctivagus* (Wied, 1820)

(*) refers to English common names as cited in the scientific literature; (**) refers to those English common names freely translated by the authors.

Most of the bird species were given a single name (even when it is a compound name such as *beija-flor* = hummingbird), although they represent one or more Linnaean species. In other cases, some species are popularly known with different popular names but they correspond to just one Linnaean species. Considering the relationship between generic taxa and scientific species, we registered the occurrence of three types of correspondence between the biological and popular classification systems proposed by Berlin et al. [5]. For instance, out of the three types of *beija-flor*, only one receives a specification, i.e. *beija-flor-rabo-de-tesoura* (*Eupetomena macroura*), due to the morphology of its tail. This type is named one-to-one correspondence. The other two species (*Phaethornis pretrei* and *Florisuga fusca*) are named only *beija-flor*, with an under-differentiation of type 2. The same happens with the popular name *garça*, which represents two scientific species (*Ardea alba* and *Bubulcus ibis*). We observed the occurrence of an over-differentiation for the specific names *coruja-amanhã-eu-vou* and *coruja-bacurau*, identified by means of vocalization, referring to a single species *Hydropsalis albicollis*. The first specific refers to a common singing in this species and is possibly related with the reproductive behavior, while the second one, *coruja-bacurau*, refers to its calling [14].

Common name formation in this study follows different criteria, such as morphology (coloring pattern, body shape and size), behavior (vocalization, reproduction, and feeding), habitat, and anthropogenic features (Table 3). However, vocalization and coloring pattern in birds were the criteria used more frequently by respondents and, according to Berlin [44], morphology is one of the main criteria used to designate ethnospecies, as well as to differentiate them.

**Table 3:** Common name formation and synonyms of bird species recorded in the village of Pedra Branca (Santa Teresinha, Bahia)

**Nomenclatural criteria**	**Common names/Synonymy**
**Morphologic**	
Color	Anu-branco, anu-preto, azulão, beija-flor-verde, gavião-pedrez, jacu-gogó-vermelho, nambu-pé-roxo, nambu-pé-vermelho, pássaro-preto, rolinha-branca, rolinha-caldo-de-feijão, sabiá-bico-de-osso, sabiá-branca, sangue-de-boi, urubu-preto, urubu-cabeça-vermelha.
Size	Bem-te-vi-menor, codorna-maior, gavião-carcará-menor, corujão.
**Body shape**	Beija-flor-rabo-de-tesoura, bem-te-vi-coroão, bigode, coleiro, codorna-pimpão, caburé-de-orelha, corujão-de-orelha, coruja-rabo-de-tesoura, charuteira.
**Habitat**	Assanhaço-coqueiro, caburé-de-estaca, caburé-de-murundu, canário-da-capoura, canário-da-terra, canário-belga, espanta-boiada, pato-d’água.
**Behavior**	
Reproduction	Cava-chão, carrega-madeira, joão-de-barro, viuvinha.
Vocalization	Acauã, bacurau, bem-te-vi, cancan, cavala, coruja-amanhã-eu-vou, coruja-bacurau, gué-gué, chorão, guriatá-vivi, jesus-meu-deus, maria-do-dia, peixe-frito, sede-sede, rolinha-fogo-apagou, saiacaia, socó-boi, tiotoin, trinca-ferro, tiziu, três-potes.
Feed	Beija-flor, chupa-laranja, papa-laranja, gavião-carrapateiro, gavião-pega-pinto, gavião-peneira, martim-pescador, mergulhão, papa-capim, pica-pau, sabiá-lasca-carne, sangue-de-boi, urubu-rei,.
**Anthropogenic aspect**	Lavandeira.

Usually, species with rather peculiar morphological similarities are not differentiated and they are given the same name [45,46], as in the cases of *corujão* (*Pulsatrix koeniswaldiana; Strix virgata*), *garça* (*Ardea alba; Bubulcus ibis*), and *gavião-rapina* (*Geranoaetus albicaudatus; Buteo brachyurus*). Moreover, feather coloring patterns are morphological features also used by residents to differentiate females from males, and the latter, usually, have feathers with brighter colors, due to sexual selection, that is, the female will choose the male because of its physical features [47].

Vocalization is an important aspect for identifying bird species, that is, the sound emitted by them often becomes their popular name on a local basis. Thus, there is an onomatopoeic name formation [44,4,48,16,51].

Among behavioral criteria, also stands out name formation through trophic ecology, such as in the case of specific names: *gavião-carrapateiro* (*Milvago chimachima*), which feeds on ticks at cattle and horses; *gavião-pega-pinto* (*Rupornis magnirostris*), which feeds on chicks of other birds, among them the domestic ones, regarded as easy prey; and *gavião-peneirador* (*Elanus leucurus*), which has the habit of hovering against the wind to see its prey [14,52]. Other studies also pointed out hawk grouping as related to feeding behavior [53,45]. The ethnospecies *urubu-rei* (*Sarcoramphus papa*) was the only one related to the generic name *urubu* (*urubu-cabeça-vermelha* e o *urubu-preto*), which was named due to its feeding behavior, as the existence of a hierarchy between these species has been reported, where other species feed only after *urubu-rei *[14].

Regarding names related to reproductive behavior, 3 ethnospecies were related to nest formation: *cava-chão* (*Nystalus maculatus*), which digs holes in slopes; *carrega-madeira* (*Phacellodomus rufifrons*), which piles wood fragments; and *joão-de-barro* (*Furnarius rufus*), which uses mud [14,54].

*Fluvicola negenta* has its nomenclatural formation based on the anthropogenic aspect. Culturally, this ethnospecies is related to religious belief, something which defines its importance within the community [16,55], as reported in the following excerpt: “*People have to say they gave the name ‘lavandeira’ because it helped Our Lady to wash clothes*” (Mrs. M, 63 years).

There are species designated on a local basis, however, they may be given another name, such as in the case of *Vanellus chilensis*, which in Pedra Branca is named *espanta-boiada*, but it is also known as *quero-quero*: “*Each region gives its name. Here it is named ‘espanta-boiada’. It is also observed in the football field. There, it is called ‘quero-quero’* ” (Mr. E, 48 years). Stauber et al. [56] emphasize the importance of studies and records of regional and local variations in the popular nomenclature used, before this is overcome by the academic nomenclature and even by the media.

In what concerns the field of ornithology, this study corroborates the importance of researching on local names [16], as a new distribution in the occurrence of *Strix virgata* has been recorded for the state of Bahia, namely at the Serra da Jiboia. This species has few records in Bahia and in accordance with literature its distribution was just known to the south of this northeastern state [57,14,59]. The latest photographic records are documented in the site Wikiaves [60-62]. In the present study this record just happened because this bird gets the popular name *corujão*, which also refers to two academic species, *Pulsatrix koeniswaldiana* and *Tyto alba*, and taking into account the morphological resemblance to the first one, *Strix virgata* was only identified through the collection of a specimen by means of some informers’ reports about its position inside the woods. Similar situation was experienced by Sick et al. [63] in the Raso da Catarina (Bahia) when they reported that the participation of local informants was decisive for the record *Anodhorhyncus leari.*

### Ethnotaxonomic classification

For starting the identification of ethnospecies by informants, we defined two lexemes: *ave* and *pássaro* (=*passarinho*). According to local perception, presence of feathers, presence of wings, being able to fly, as well as body size and breeding for slaughter, were relevant features for this differentiation. The term *ave* mainly refers to species bred at home, such as *galinha* (*Gallus gallus*), *pato* (*Cairina moschata*), and *sacué* (*Numida meleagris*). In turn, *pássaro* or *passarinho* are terms used to designate species that are not domesticated, even those bred in captivity. A testimony exemplifies this semantic distinction: “*‘Ave’ is bred at home and ‘pássaro’ always lives in another place. ‘Galinha’, ‘peru’ are called ‘aves’ and the animals we see flying out there are called ‘pássaros’*” (Mr. R, 69 years).

The presence of feathers leads some informants to include the term *pássaro* into *ave*, but it is differentiated due to the species habitat, either on trees or on the ground: “*All feathered ‘pássaros’ are called ‘ave’, you know. All of them are this kind of ‘ave’. The difference is subtle, because the difference of ‘galinha’ is that it lives on the ground, and ‘passarinho’ does not, it lives over there, it flies, it also sits on the floor, but ‘galinha’ lives on the ground. ‘Passarinhos’ build their nests on trees and ‘galinhas’ on the floor*” (Mr. J, 48 years).

Other studies have also reported these categories, and the ethnospecies *galinha*, *peru*, and *periquito* are included as “bred at home” or “bird on the ground”, as *gavião*, *garça*, and *pássaro-preto* are “wild bird”, “self-bred bird”, or “flying bird”, and these categories include species that are called *pássaros* or *passarinhos*, due to captive breeding [64-66].

For some communities, this category of *aves* covers animals that fly, present a beak, feathers, and lay eggs [19,16]. Brown [67] introduces *ave* as large animals, which have wings, feathers, and a beak (always including *pássaros*), whereas the bird life form includes flying mammals, such as *morcegos*, a fact also found out by Jensen [19] and Blumer [68]. This inclusion is registered in Pedra Branca, as observed in the following interview excerpt: “*I think ‘morcego’ is regarded as ‘passarinho’, it has wings and flies*” (Mr. F, 31 years).

Ethnotaxonomic information of respondents seemingly allows us to order them hierarchically, according to the principles of categorization proposed by Berlin [4], where three hierarchical levels were recognized: life form, generic, and specific. In this study, the lexemes *pássaro* and *passarinho* match the ethnotaxonomic level “life form”. These lexemes include all wild birds cited in the study, but they do not correspond to the Linnaean taxonomy, because *pássaros* are animals belonging only to the Passeriformes order [69,70].

**Figure 2:**
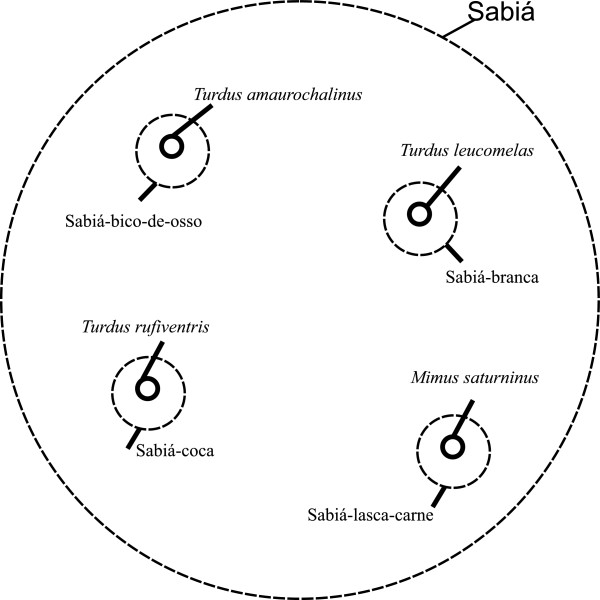
Specific folk generic “sabiá” and its equivalents in academic taxonomy.

The recognition and grouping of the generic name *sabiá* in the region, for instance, mainly relies on morphology, and the vocalization and trophic criteria are responsible for identifying and defining the specific names (Figure 2). Even though sounds are similar, we can identify species in the field [14,53]. Grouping the specific names *sabiá-bico-de-osso*, *sabiá-branca*, and *sabiá-coca* corresponds to the Turdidae family in the Linnaean taxonomy, while the specific name *sabiá-lasca-carne* belongs to the Linnaean Mimidae family. The correspondences found were 1:1 for the 4 specific names cited.

**Figure 3:**
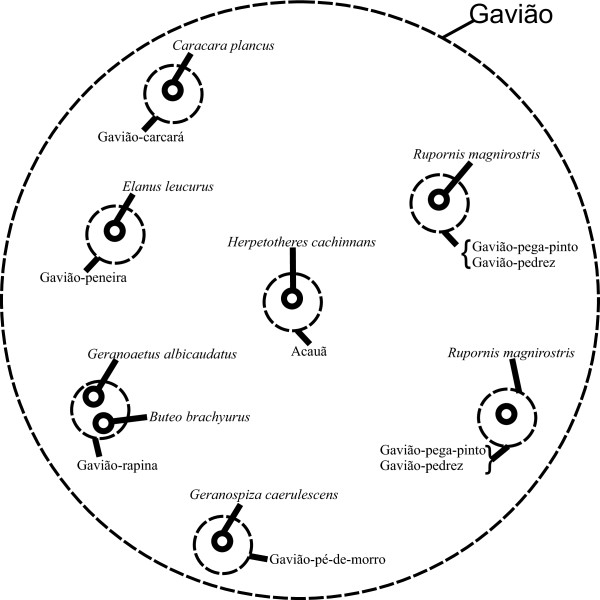
Specific folk generic “gavião” and its equivalents in academic taxonomy.

According to respondents’ perception, *acauã* (*Herpetotheres cachinnans*) is recognized as a kind of *gavião*, but it is not included in this generic name, constituting a monotypic generic name. The generic name *gavião* has eight specific names and the morphological and behavioral criteria are used to group this set of birds (Figure 3). The specific names *gavião-carcará* (*Caracara plancus*), *gavião-carrapateiro* (synonym *gavião-carcará-menor*, *Milvago chimachima*), and *acauã* (*H. cachinnans*) correspond to the Falconidae family, and the other ones belong to the Accipitridae family. In this example, 4 1:1 correspondences, 2 under-differentiations, and 1 type B over-differentiations are found.

### Final remarks

The information registered here shows a broad ethno-ornithological knowledge within the community living in the village of Pedra Branca, so that many birds are given names followed by synonyms, while others have only a single name and there is a correspondence between the folk ethnotaxonomy and Linnaean taxonomy.

The results reinforce the need of the participation of local informants in inventories of birds in order to record potential new distributions in the occurrence of species, mainly those nocturnal and even migratory birds.

Through the analysis of popular names, it was possible to identify the nomenclatural criteria used to designate the birds; vocalization and coloring pattern were those more frequently used. Virtually all respondents reported the same etymology for the common names of ethnospecies, something which means that local names are strongly conveyed within the community.

Feeding behavior was a relevant aspect in this research, because besides forming names, it also allowed identifying and hierarchically ordering species. However, the formation of some names did not follow any criteria, as some ethnospecies were identified due to some kind of behavior, vocalization, and habitat.

According to the Berlinean classification model, there was a hierarchy into three levels: life form, generic, and specific. Even meeting two lexemes *ave* and *pássaro* (*passarinho*), we opted to use *pássaro* at the life form level, listing only the wild birds.

## Competing interests

The authors declare that they have no competing interests.

## Authors’ contributions

ATGL carried out the field research and drafted the manuscript. EMCN, CGM, and FMF participated in its design and coordination, and helped to draft the manuscript. All authors read and approved the final manuscript.
